# Coronary plaque progression of non-culprit lesions after culprit percutaneous coronary intervention in patients with moderate to advanced chronic kidney disease: intravascular ultrasound and integrated backscatter intravascular ultrasound study

**DOI:** 10.1007/s10554-015-0633-y

**Published:** 2015-02-28

**Authors:** Kuninobu Kashiyama, Shinjo Sonoda, Yoshitaka Muraoka, Yoshiyuki Suzuki, Fumihiko Kamezaki, Yuki Tsuda, Masaru Araki, Masahito Tamura, Masaaki Takeuchi, Haruhiko Abe, Masahiro Okazaki, Yoshihisa Fujino, Yutaka Otsuji

**Affiliations:** 1 Second Department of Internal Medicine, School of Medicine, University of Occupational and Environmental Health, 1-1 Iseigaoka, Yahatanishi-ku, Kitakyushu, 807-8555 Japan; 2 Department of Preventive Medicine and Community Health, School of Medicine, University of Occupational and Environmental Health, Kitakyushu, Japan

**Keywords:** Chronic kidney disease, Coronary atherosclerosis, Intravascular ultrasound, Plaque characteristics

## Abstract

Previous studies have suggested that the deterioration of renal function increases the risk of major adverse clinical events not only in culprit lesions but also in non-culprit lesions (NCLs) after percutaneous coronary intervention (PCI). This study evaluated serial coronary plaque change of NCL in patients with different stages of chronic kidney disease (CKD) using intravascular ultrasound (IVUS) and integrated backscatter IVUS (IB-IVUS). In 113 patients (113 NCLs) underwent both IVUS-guided PCI and follow-up IVUS, volumetric IVUS analyses were performed at proximal reference NCLs in de novo target vessels post PCI and at 8-month follow-up. NCLs were divided into 4 groups based on baseline CKD stage: CKD-1, n = 18; CKD-2, n = 42; CKD-3, n = 29; and CKD4–5, n = 24. We compared serial changes of plaque burden and composition among groups under statin treatment. Plaque progression occurred in CKD-3 (+4.6 mm^3^, *p* < 0.001) and CKD4–5 (+9.8 mm^3^, *p* < 0.001) despite anti-atherosclerotic treatment, whereas plaque regression occurred in CKD-1 (−5.4 mm^3^, *p* = 0.002) and CKD-2 (−3.2 mm^3^, *p* = 0.001) mainly due to initiate statin treatment after PCI. Plaque volume change was correlated with eGFR (*p* < 0.0001). Multivariate analysis showed CKD stage 3–5 was an independent predictor of plaque progression. Regarding IB-IVUS analyses, lipid plaque increased in CKD-3 (+4.6 mm^3^, *p* < 0.001) and CKD4–5 (+5.4 mm^3^, *p* < 0.001), but decreased in CKD-2 (−2.7 mm^3^, *p* < 0.05). Fibrotic plaque also increased in CKD4–5 (+3.4 mm^3^, *p* < 0.001). Moderate to advanced CKD was associated with coronary plaque progression characterized by greater lipid and fibrotic plaque volumes in NCL under statin treatment after culprit PCI.

## Introduction

It is well known that deteriorating renal function is associated with increasing cardiovascular events in patients with chronic kidney disease (CKD) [1–3]. Several studies suggested that the deteriorating renal function increases the risk of major adverse clinical events not only in culprit lesions but also in non-culprit lesions (NCLs) after percutaneous coronary intervention (PCI) [4–6]. Conventional intravascular ultrasound (IVUS) can identify the amount of coronary plaque, and integrated backscatter (IB) IVUS can assess the plaque compositions such as lipid, fibrous tissue, dense-fibrous tissue, and calcification with a high sensitivity and specificity compared to histology [7–9]. Previous virtual histology (VH) IVUS studies showed that fibrous volume decreased and dense-calcium volume increased as renal function decreased in either culprit lesions or NCLs, although plaque volume was comparable among patients with varying levels of renal function [10]. An IB-IVUS study in NCLs showed that plaque volume was greater in CKD patients than in non-CKD patients, and that there was a negative correlation between lipid volume and the estimated glomerular filtration rate (eGFR) and a positive correlation between fibrous volume and eGFR [11]. To prevent procedural complications and secondary cardiovascular events, patients are offered statin treatment after PCI. Although statin is effective in reducing cardiovascular events in patients with mild to moderate CKD [12], such an effect remains to be clarified in patient with advanced CKD.

This study assessed the relationship between CKD stage and coronary plaque change in NCLs using conventional IVUS in patients with CKD, including advanced CKD, under statin treatment after culprit PCI. In addition, we assessed the relationship between CKD stage and plaque characteristics in NCLs using IB-IVUS as a subgroup analysis.

## Methods

### Study design and patients

This study was a retrospectively planned observational study for non-culprit coronary lesions in patients with CKD who underwent IVUS-guided PCI.

**Fig. 1:**
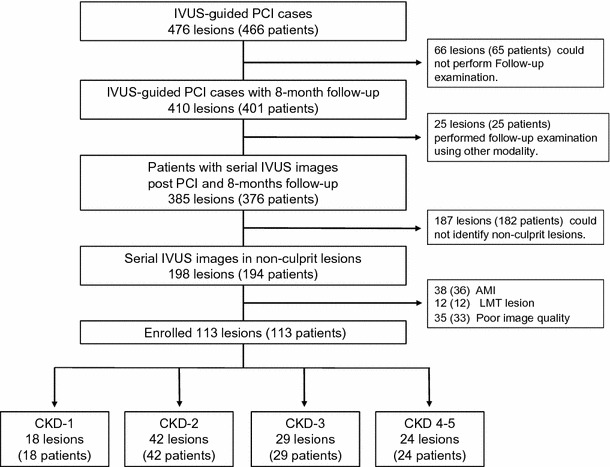
Outline of the breakdown of patients. *LMT* left main trunk, *NCL* non-culprit lesion

**Fig. 2:**
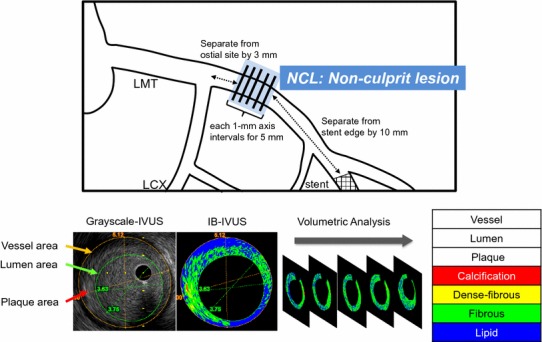
Definition of NCL and the method of IVUS measurement

Figure 1 shows a breakdown of patients included in this study. Between April 2010 and March 2014, we had 543 consecutive PCI cases. IVUS-guided PCI was performed for 476 lesions (466 patients) in this period. After 8 months of standard medical treatment including statin, follow-up coronary angiography and IVUS examination were performed for 410 lesions (401 patients), irrespective of the presence or absence of symptoms. After excluding patients who had undergone follow-up examination using other modalities (25 patients with 25 lesions), serial IVUS images for 385 lesions (376 patients) obtained at both baseline and follow-up using identical IVUS catheters (ViewIT™, Terumo, Tokyo, Japan, or Atlantis™ SR Pro 2, Boston Scientific, Natick, MA, USA) were available for analysis. A target NCL in this study was defined as 5-mm segment which was 3 mm distal from ostium of each coronary artery (LAD, LCX, RCA), with percent diameter stenosis <50 % on quantitative coronary angiography (QCA). And also, a target NCL must be at least 10-mm proximal to the stented segment to remove stent edge effects (Fig. 2). Therefore, additional 187 lesions (182 patients) were excluded because we could not identify NCLs. After excluding 36 patients (38 lesions) with acute myocardial infarction, 12 patients (12 lesions) with left main trunk lesions, and 33 patients (35 lesions) with poor IVUS image quality, we enrolled 113 patients (113 NCLs) in this study. The patients were classified into 4 groups based on CKD stage: CKD-1: eGFR ≧90 ml/min/1.73 m^2^; CKD-2: eGFR <90 and ≧60 ml/min/1.73 m^2^; CKD-3: eGFR <60 and ≧30 ml/min/1.73 m^2^; and CKD4–5: eGFR <30 ml/min/1.73 m^2^ with/without hemodialysis. The eGFR was calculated using the Diet in Renal Disease equation modified with the Japanese coefficient: eGFR (ml/min/1.73 m^2^) = 194 × serum creatinine (mg/dl)^−1.094^ × age (years)^−0.287^ (× 0.739 for female patients).

Standard medical treatment including statin was performed during the 8-month follow-up. Medication was left to the discretion of the treating physician. Dyslipidemia was controlled to the levels of low-density lipoprotein (LDL) cholesterol <100 mg/dl and high-density lipoprotein (HDL) cholesterol >40 mg/dl. All patients were prescribed statins (rosuvastatin, atorvastatin, pitavastatin or pravastatin) to manage the lipid profile to the target control level, unless contraindicated. Diabetes mellitus was controlled to HbA1c level <7.0 %. Hypertension was controlled to the systolic blood pressure (SBP) <130 mmHg and diastolic blood pressure (DBP) <80 mmHg. A study protocol was approved by the appropriate institutional review committee, and informed consent was obtained from all patients.

### IVUS imaging

After the administration of 1–2 mg of intracoronary isosorbide dinitrate, IVUS images were obtained from the distal site of the culprit lesion using a 40-MHz IVUS catheter (ViewIT™ or Atlantis SR Pro 2™) attached to an IVUS imaging system (VISIWAVE™ or iLab™) with a motorized pullback at 0.5 mm/s. IB-IVUS images were obtained using software supplied with 1 of 2 IVUS machines (VISIATRAS™, Terumo, Japan).

Eight months later, a follow-up IVUS examination was repeated in the same coronary segment imaged at the baseline examination, regardless of clinical symptoms. Corresponding images were identified by the distance from the fiduciary side branches.

### Measurements of conventional and IB-IVUS parameters

Each conventional IVUS and IB-IVUS parameter were measured at baseline and after 8 months. One experienced investigator who was unaware of the patient group allocation performed the quantitative IVUS analysis.

IVUS analyses were performed according to the criteria described in the American College of Cardiology Clinical Expert Consensus document on IVUS [13]. Cross-sectional lumen area, cross-sectional vessel area within the external elastic membrane, and plaque area (external elastic membrane area minus lumen area) were determined using software attached to each IVUS machine (VISIATRAS or EchoPlaque™, Indec System, Mountain View, CA, USA).

The definition of IB parameters for each histological category were determined by comparing histologic images reported in the previous study [14]. Plaque properties were diagnosed into 4 types according combining spectral parameters of posterior scattering signal: lipid; fibrous; dense-fibrous; or calcification. The area of each component was automatically measured in each plaque.

The volume of each conventional IVUS and IB-IVUS parameters for 5-mm length in the NCL was calculated using integration. A representative case is shown in Fig. 2.

### Statistical analysis

All data are expressed as the mean ± SD. Differences in continuous variables were compared using the paired *t* test. Differences among 4 groups were assessed with analysis of variance (ANOVA) for independent samples and the Chi square test for comparison of categorical variables, and with an unpaired Student’ t-test for continuous variables. Plaque volume change was examined using analysis of covariance (ANCOVA), with the baseline value as a covariate and grouping variable (CKD stage) as a factor. Characteristics at baseline with an imbalance between groups were also included in an ANCOVA model. Correlations between eGFR and plaque volume change were analyzed using a simple linear regression analysis. To identify predictors of plaque progression, simple and multiple regression analyses were performed. The association between CKD stage and coronary plaque change after adjusting for conventional risk factors and medications is summarized as an OR (odds ratio) estimated by multivariate logistic regression analysis.

A *p* value <0.05 was considered to indicate statistical significance. Statistical analysis was performed using JMP version 9.0 (SAS, Cary, NC, USA) and Stata version 13.1 (StataCorp LP, College Station, TX, USA).

## Results

### Study populations

A total of 113 NCLs were measured in 113 patients without significant stenosis. NCLs were categorized as follows: CKD-1, 18 lesions; CKD-2, 42 lesions, CKD-3, 29 lesions, CKD4–5, 24 lesions (Fig. 1).

### Baseline clinical characteristics

Table 1 shows baseline clinical characteristics. Age in CKD-3, unstable angina pectoris (UAP) and DBP in CKD-1, and diabetic patients in CKD4–5 were higher than the other groups. Otherwise, there were no significant differences in other parameters among the groups.

**Table 1:** Baseline clinical characteristics

	CKD-1 (n = 18)	CKD-2 (n = 42)	CKD-3 (n = 29)	CKD4–5 (n = 24)	*p* value
Age	61 ± 12	70 ± 9	77 ± 9	69 ± 9	<0.0001
Male sex	16 (89)	27 (64)	20 (69)	18 (75)	0.2
BMI (kg/m^2^)	23.4 ± 3.6	24.3 ± 3.3	23.9 ± 3.6	22.2 ± 3.4	0.1
Unstable angina pectoris	10 (56)	10 (24)	6 (21)	1 (4)	0.001
Hypertension	13 (72)	32 (76)	22 (76)	20 (83)	0.8
Hyperlipidemia	8 (44)	26 (62)	13 (45)	16 (67)	0.2
Diabetes mellitus	7 (39)	11 (26)	13 (45)	18 (75)	0.002
Current smoking	7 (39)	11 (26)	9 (31)	10 (42)	0.6
Baseline biochemistry data					
LDL cholesterol (mg/dl)	113 ± 35	105 ± 35	101 ± 29	101 ± 38	0.6
HDL cholesterol (mg/dl)	52 ± 11	54 ± 14	56 ± 15	47 ± 13	0.1
HbA1c (%)	6.3 ± 1.2	6.3 ± 1.1	6.2 ± 0.7	6.6 ± 1.2	0.6
hs CRP (mg/dl)	0.5 ± 0.9	0.7 ± 1.9	0.4 ± 0.9	0.6 ± 1.6	0.8
SBP (mmHg)	137 ± 22	130 ± 19	133 ± 19	140 ± 22	0.2
DBP (mmHg)	84 ± 15	72 ± 14	74 ± 13	75 ± 14	0.03
Follow-up period (months)	7.2 ± 1.9	8.0 ± 1.7	7.8 ± 1.3	8.1 ± 1.9	0.3
Lesion location (%)					
LAD/LCX/RCA	55/17/28	60/5/36	52/31/17	46/13/42	0.07

Data are expressed as mean ± SD or number (%), or percent

*LAD* left anterior descending artery, *LCX* left circumflex artery, *RCA* right coronary artery

### Risk factor control

The anti-atherosclerotic medications use and the degree of risk factor control at follow-up are summarized in Table 2. Statin treatment was prescribed for more than 80 % of each group. The patients with statin treatment initiated before PCI (continue statin group: CS) included 22 % in CKD-1, 33 % in CKD-2, 34 % in CKD-3, and 46 % in CKD4–5. The patients with statin treatment initiated after PCI (initiate statin group: IS) included 61 % in CKD-1, 62 % in CKD-2, 55 % in CKD-3, and 42 % in CKD4–5. The frequencies of CS and IS were comparable among 4 groups. Beta-blockers were more likely to be used in CKD-3 and CKD4–5. CKD4–5 had significantly higher HbA1c levels at follow-up, and CKD-3 and CKD4–5 had significantly higher SBP than CKD-1 and CKD-2. The achievement ratio of the HbA1c target level in the CKD4–5, and the target SBP in CKD-3 and CKD4–5 were significantly lower than those in the other groups.

**Table 2:** Risk factor management at follow-up

	CKD-1	CKD-2	CKD-3	CKD4–5	*p* value
Medications after PCI					
Statin	15 (84)	40 (95)	26 (90)	21 (85)	0.24
(Continue statin group: CS)	4 (22)	14 (33)	10 (34)	11 (46)	0.45
(Initiate statin group: IS)	11 (61)	26 (62)	16 (55)	10 (42)	0.79
β-blocker	8 (44)	12 (29)	18 (62)	12 (50)	0.04
DAPT	60 (100)	33 (100)	20 (100)	20 (100)	–
ACE-I/ARB	13 (72)	25 (60)	20 (69)	17 (71)	0.7
Clinical data at follow-up					
LDL cholesterol (mg/dl)	96 ± 19	87 ± 26	81 ± 18	87 ± 34	0.3
HDL cholesterol (mg/dl)	54 ± 11	55 ± 14	55 ± 15	47 ± 13	0.1
HbA1c (%)	6.3 ± 1.2	6.2 ± 1.0	6.2 ± 0.7	6.9 ± 1.3	0.03
CRP (mg/dl)	0.2 ± 0.2	0.2 ± 0.9	0.1 ± 0.1	0.3 ± 0.4	0.6
SBP (mmHg)	120 ± 15	119 ± 13	130 ± 23	133 ± 20	0.008
DBP (mmHg)	72 ± 15	67 ± 10	73 ± 15	71 ± 12	0.3
Change from baseline					
LDL cholesterol (mg/dl)	−17 ± 37	−19 ± 33	−20 ± 28	−15 ± 46	0.9
HDL cholesterol (mg/dl)	+2 ± 7	+1 ± 10	0 ± 9	0 ± 11	0.9
HbA1c (%)	0 ± 0.4	0 ± 0.8	0 ± 0.7	+0.4 ± 0.8	0.06
CRP (mg/dl)	−0.4 ± 0.7	−0.4 ± 1.5	−0.2 ± 0.9	−0.3 ± 1.6	0.9
SBP (mmHg)	−16 ± 23	−10 ± 22	−3 ± 24	−8 ± 32	0.3
DBP (mmHg)	−12 ± 15	−5 ± 14	−1 ± 13	−4 ± 16	0.1
Achievement ratio of target control level at follow-up					
LDL cholesterol <100 mg/dl	10 (55)	31 (74)	24 (83)	16 (67)	0.2
HDL cholesterol >40 mg/dl	17 (94)	37 (88)	22 (76)	17 (71)	0.1
HbA1c <7.0 %	15 (83)	39 (93)	24 (83)	12 (50)	0.03
SBP <140 mmHg	16 (89)	40 (95)	22 (76)	17 (71)	0.03
DBP <80 mmHg	12 (67)	36 (86)	21 (72)	20 (83)	0.3

Data are expressed as mean ± SD or number (%)

Continue statin group: CS, patients with statin treatment have already initiated before PCI

Initiate statin group: IS, patients with statin treatment initiated after PCI

*DAPT* dual antiplatelet therapy, *ACE-I* angiotensin converting enzyme inhibitor, *ARB* angiotensin II receptor blocker

### Conventional IVUS analysis

**Fig. 3:**
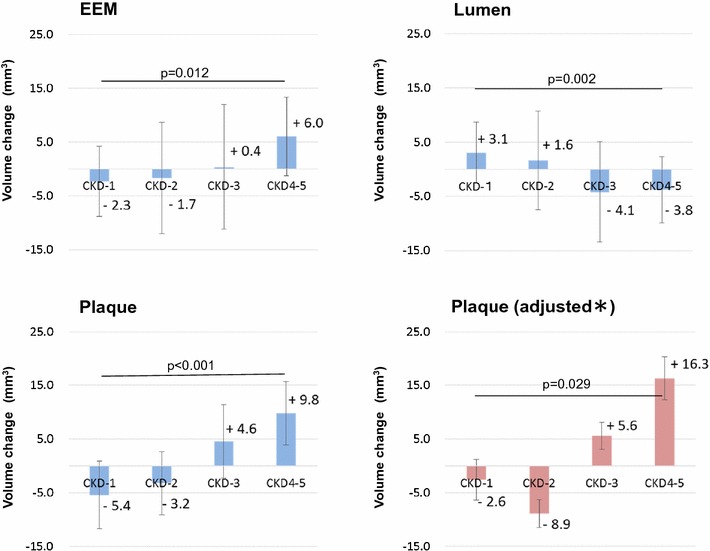
Comparison of volume change data using gray-scale IVUS data at baseline and follow-up. *Plaque volume adjusted on ANCOVA for differences in clinical characteristics including baseline plaque volume, age, gender, BMI, UAP, current smoking, HT, DL, DM, follow-up LDL cholesterol and follow-up HDL cholesterol levels, and the use of ACE-I/ARB, beta-blocker, and statin

**Fig. 4:**
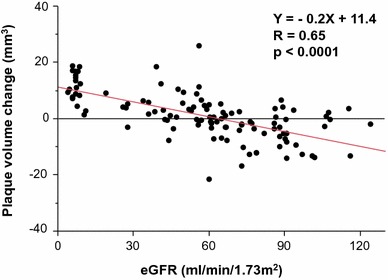
Correlations between the eGFR at baseline and plaque volume change

Table 3 shows the volume data using conventional gray-scale IVUS. The EEM volume was significantly greater in CKD4–5 than the other groups at both baseline and follow-up. The lumen volume showed no significant differences among groups at baseline; however, at follow-up, it was significantly greater in CKD-1. The plaque volume was significantly greater in CKD4–5 than the other stages at both baseline and follow-up. The EEM volume significantly increased in CKD4–5, whereas it did not in the other groups. The lumen volume significantly decreased in CKD-3 and CKD4–5, but increased in CKD-1. The plaque volume significantly increased in CKD-3 and CKD4–5, whereas it significantly decreased in CKD-1 and CKD-2. Furthermore, in the ANCOVA models including age, gender, BMI, unstable angina pectoris, hypertension, hyperlipidemia, diabetes, follow-up LDL-cholesterol and HDL-cholesterol levels, ACE-I/ARB use, beta-blocker use, and statin use, did not change the results, reflected by still greater plaque progression in moderate to advanced CKD patients (Fig. 3). Univariate linear regression analysis showed the baseline eGFR correlated significantly with plaque volume change (R = 0.65, *p* < 0.0001) (Fig. 4). On univariate and multivariate logistic regression analyses, after considering CKD stage, confounding factors (age, gender, and unstable angina pectoris), coronary risk factors and medications, CKD stage 3–5 demonstrated the strongest association with plaque progression (Tables 4, 5).

**Table 3:** Parameters evaluated using gray-scale IVUS

Volume (mm^3^)	CKD-1	CKD-2	CKD-3	CKD4–5	*p* value
EEM					
Baseline	88.8 ± 21.3	80.0 ± 23.3	78.2 ± 21.1	93.1 ± 19.7^c,e^	0.039
Follow-up	86.5 ± 24.5	78.3 ± 24.2	79.6 ± 16.0	99.1 ± 22.9^d,f^	0.002
Lumen					
Baseline	54.2 ± 15.3	44.0 ± 19.7	42.5 ± 17.5	47.4 ± 13.2	0.121
Follow-up	57.3 ± 18.0	45.6 ± 20.1^a^	38.4 ± 12.1^b^	43.6 ± 15.8^a^	0.004
Plaque					
Baseline	34.7 ± 14.2	35.9 ± 12.1	35.6 ± 9.1	45.8 ± 16.3^b,d,e^	0.009
Follow-up	29.2 ± 11.9	32.7 ± 11.1	40.2 ± 7.9^b,c^	55.6 ± 19.3^b,d,f^	<0.001

Data are expressed as mean ± SD

Vs. CKD-1: ^a^ *p* < 0.05, ^b^ *p* < 0.01. Vs. CKD-2: ^c^ *p* < 0.05, ^d^ *p* < 0.01. Vs. CKD-3: ^e^ *p* < 0.05, ^f^ *p* < 0.01

**Table 4:** Univariate logistic regression analysis estimates for plaque progression

Variable	Odds ratio	95 % CI	*p* value
CKD stage 1	Reference		
CKD stage 2	0.95	0.25–3.62	0.945
CKD stage 3–5	15.1	4.07–55.6	<0.0001
Age	1.02	0.98–1.06	0.300
Gender	1.38	0.61–3.15	0.438
BMI	0.97	0.87–1.07	0.513
UAP	0.26	0.10–0.69	0.007
Current smoking	1.11	0.51–2.44	0.790
Follow-up SBP >140 mmHg	1.02	1.00–1.04	0.122
Follow-up LDL >100 mg/dl	1.00	0.98–1.01	0.737
Follow-up HbA1c >7.0 %	1.56	1.04–2.34	0.032
ACE-I/ARB use	0.71	0.32–1.55	0.389
Β-blocker use	2.13	1.00–4.55	0.049
Statin use	0.53	0.15–1.92	0.332

**Table 5:** Multivariate logistic regression analysis included only variables that showed significant association in univariate analyses

Variable	Odds ratio	95 % CI	*p* value
CKD stage 1	Reference		
CKD stage 2	0.93	0.21–4.14	0.928
CKD stage 3–5	13.56	2.85–64.44	0.001
Age	0.98	0.94–1.04	0.631
Gender	1.67	0.55–5.11	0.367
UAP	0.39	0.11–1.32	0.129
Follow-up HbA1c >7.0 %	1.36	0.87–2.12	0.178
Β-blocker use	1.38	0.51–3.70	0.524

### IB-IVUS analysis

Serial IB-IVUS images were acquired from 93 patients with 93 NCLs: CKD-1, 15 lesions; CKD-2, 33 lesions; CKD-3, 23 lesions; CKD4–5, 22 lesions. Baseline clinical characteristics and risk factor control were similar to gray-scale IVUS analysis.

In IB-IVUS analyses, although percent calcification was larger in CKD4–5 than the other groups at baseline, there were no significant differences in percent of each component (relative values) among groups at follow-up. However, the volumes of calcification, dense-fibrosis, and lipid were significantly greater in CKD4–5 than the other groups at both baseline and follow-up. Although fibrous volume showed no significant difference among groups at baseline, it was greater in CKD4–5 than the other groups at follow-up (Table 6).

**Table 6:** Parameters evaluated using IB-IVUS

Volume	CKD-1 (n = 15)	CKD-2 (n = 33)	CKD-3 (n = 23)	CKD4–5 (n = 22)	*p* value
Calcification					
Baseline					
%	1.0 ± 1.5	1.2 ± 1.3	1.5 ± 1.6	2.5 ± 2.4^b,d^	0.02
mm^3^	0.3 ± 0.4	0.4 ± 0.6	0.6 ± 0.9	1.3 ± 1.5^b,d,f^	0.002
Follow-up					
%	1.1 ± 2.0	1.8 ± 2.4	1.5 ± 1.7	2.4 ± 2.2	0.33
mm^3^	0.3 ± 0.5	0.6 ± 0.7	0.6 ± 0.6	1.6 ± 1.8^b,d,f^	<0.001
Dense-fibrous					
Baseline					
%	3.5 ± 1.9	3.8 ± 2.4	5.1 ± 3.1	5.0 ± 2.3	0.10
mm^3^	1.3 ± 0.8	1.3 ± 0.8	1.8 ± 1.2	2.4 ± 1.8^b,d^	0.005
Follow-up					
%	3.8 ± 3.0	4.7 ± 3.3	4.3 ± 2.5	5.1 ± 2.4	0.55
mm^3^	1.1 ± 0.9	1.5 ± 1.3	1.7 ± 1.1	3.1 ± 2.1^b,d,f^	<0.001
Fibrous					
Baseline					
%	45.0 ± 8.7	38.7 ± 11.2	43.5 ± 10.1	39.2 ± 8.3	0.12
mm^3^	16.1 ± 7.2	13.4 ± 5.1	15.7 ± 5.2	17.3 ± 4.7^c^	0.066
Follow-up					
%	43.6 ± 9.8	40.6 ± 11.5	37.6 ± 11.2	38.3 ± 9.9	0.34
mm^3^	12.8 ± 4.8	12.8 ± 5.9	14.9 ± 5.9	20.7 ± 6.3^b,d,f^	<0.001
Lipid					
Baseline					
%	50.5 ± 10.6	54.2 ± 12.9	49.9 ± 12.4	53.4 ± 8.4	0.53
mm^3^	18.3 ± 8.3	20.2 ± 9.7	18.0 ± 6.5	25.7 ± 10.5^a,c,f^	0.024
Follow-up					
%	51.5 ± 12.6	53.0 ± 14.5	56.6 ± 13.0	54.2 ± 10.4	0.65
mm^3^	15.8 ± 9.6	17.5 ± 8.7	22.6 ± 7.1^a^	31.1 ± 12.6^b,d,f^	<0.001

Data are expressed as mean ± SD

Vs. CKD-1: ^a^ *p* < 0.05, ^b^ *p* < 0.01. Vs. CKD-2: ^c^ *p* < 0.05, ^d^ *p* < 0.01. Vs. CKD-3: ^e^ *p* < 0.05, ^f^ *p* < 0.01

**Fig. 5:**
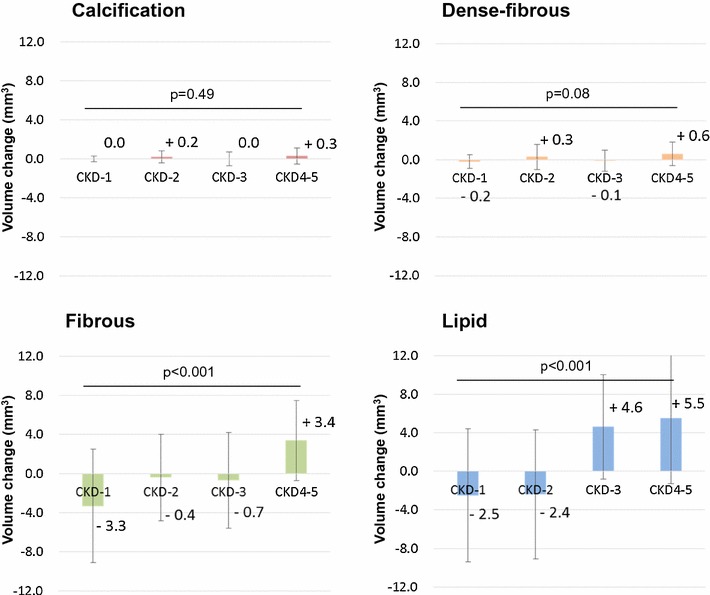
Comparison of volume change in plaque characteristics using IB-IVUS data at baseline and follow-up

**Fig. 6:**
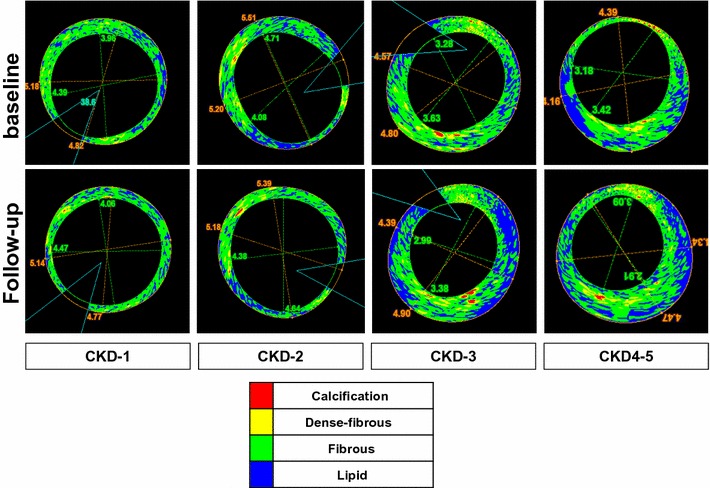
Representative IB-IVUS image of various CKD stages at baseline and follow-up. *Red* calcification, *yellow* dense-fibrous, *green* fibrous, *blue* lipid. Plaque decreased in CKD-1 and CKD-2, whereas it increased in CKD-3 and CKD4–5. Fibrous tissue increased in CKD4–5, but decreased in CKD-1. Lipid increased in CKD-3 and CKD 4–5, whereas decreased in CKD-1 and CKD-2

The calcification and dense-fibrosis showed no significant changes in all groups during 8 months of follow-up. Fibrous plaque decreased in CKD-1, but increased in CKD4–5. Lipid volume significantly increased in CKD-3 and CKD4–5 during follow-up, although it decreased in CKD-2 and CKD-3 (Fig. 5). Representative cases are shown in Fig. 6.

## Discussion

We found important findings in changes of coronary atherosclerotic plaque formation and morphology of the NCLs in each stage of CKD under standard medical treatment after PCI.

Plaque volume was significantly greater in CKD4–5 than the other stages at both baseline and follow-up. Plaque volume significantly increased in CKD-3 and CKD4–5 despite anti-atherosclerotic treatment, but significantly decreased in CKD-1 and CKD-2 mainly due to initiate statin treatment after PCI. The multivariate regression analysis showed that CKD stage is an independent predictor of plaque progression. Regarding plaque composition, lipid plaque gain occurred in CKD-3 and CKD4–5, whereas lipid plaque regression occurred in CKD-1 and CKD-2. Fibrous plaque gain occurred in CKD4–5 only.

### Plaque progression in CKD patients

CKD is well known to be associated with high mortality resulting from coronary artery disease (CAD) compared to mortality from progression to end-stage renal dysfunction. Many previous studies showed that CKD patients have severe atherosclerosis, and that they are at high risk for CAD [15–17]. A previous study that included VH-IVUS analysis demonstrated that plaque volumes in NCLs were comparable among patients with varying levels of renal function [10, 18]. On the other hand, it was also reported that plaque volumes in patients with moderate CKD were greater than those in patients with normal renal function [11]. However, those studies analyzed only 1 timeframe. In one serial IVUS analysis, Nozue et al. [19] found that patients with normal-to-mild renal dysfunction under strong statin treatment showed significant decreases in EEM volume and plaque regression. Little is known about the impact of advanced CKD on serial change in plaque volume. In the present study, plaque progression occurred in patients with moderate to advanced CKD (CKD stage 3–5), whereas plaque regression occurred in CKD-1 or CKD-2. In this study, IS included 40–50 % of all patient, and they showed approximately 20 % reduction in LDL-cholesterol level. In CKD1–2, plaque regressions in IS were significantly greater than those in CS (6.5 ± 1.7 mm^3^ in IS vs. 2.2 ± 0.8 mm^3^ in CS, *p* = 0.01). Therefore, it is suggested that plaque regression in CKD-1 and CKD-2 was mainly due to initiate statin treatment after PCI. Previous study reported that plaque regression after newly initiated statin therapy in patients with normal to mild renal dysfunction was 1.3 (±9.1) % [19]. In the present study, plaque regressions of IS in CKD1–2 were greater compared with the previous study. It is suggested that the difference may be due to the patient characteristics. Our study included patients with mildly impaired renal function as well as those with advanced CKD, our results may suggest that plaque regression during standard medical treatment gradually decelerated with decreasing renal function. A previous randomized study failed to demonstrate clinical benefit of the rosuvastatin in patients with hemodialysis [20]. Results of the present study may support the results. An ANCOVA model and multivariate logistic model considering confounding factors, coronary risk factors, and medications, demonstrated that CKD stage have the strong association with plaque volume change. As for the multivariate logistic regression analysis, we integrated CKD-3 with CKD4–5 to perform plausible multivariate analysis. In this analysis, moderate to advanced CKD was definitely a strong independent predictor of plaque progression. Strict risk management for plaque regression is necessary, especially in patients with moderate to advanced CKD after PCI. In addition, we did not observe any clinical events associated with NCLs during this study period. Longer follow-up is needed to confirm the relationship between cardiovascular events and CKD stages.

### Impact of CKD on coronary plaque characteristics

A previous postmortem study showed that a lower eGFR is associated with a high percentage of advanced coronary atherosclerosis, defined as American Heart Association type IV (atheroma), type V (fibro-atheroma), and type VI (complicated plaque) [21]. Moreover, Miyagi et al. [8] found renal dysfunction to be significantly associated with an increased percentage of lipid volume and a decreased percentage of fibrous volume in the non-target coronary lesions of 89 patients using IB-IVUS. In addition, Hayano et al. [11] demonstrated greater lipid and smaller fibrous volumes in NCLs in a series of 201 patients using IB-IVUS. In the present study, we identified several differences in atherosclerotic plaque composition among patients with various stages of CKD. Lipid plaque regression occurred in CKD-1 and CKD-2, whereas lipid plaque gain occurred in CKD-3 and CKD4–5. Fibrous plaque also increased in CKD4–5. We speculate that the reason for these findings is the inclusion of patients who were on hemodialysis. A previous autopsy study showed that coronary plaques in hemodialysis patients are characterized by increased media thickness [17]. This finding might reflect fibrous plaque gain in hemodialysis patients during the follow-up period. The relationship between coronary plaque characteristics and mineral disorder in patients with end-stage CKD should be analyzed. Large lipid cores are considered histologic markers for plaque vulnerability, which is directly related to the risk of plaque rupture [22, 23]. Therefore, large lipid cores are considered one of the reasons for the increased risk of cardiovascular events in patients with CKD.

## Study limitations

First, the present study included a relatively small number of patients at a single center, raising the possibility of selection bias. In addition, we exclude patients with acute myocardial infarction because it is difficult to differentiate between lipid rich tissue and thrombus by IB-IVUS. Second, we defined a NCL as a 5-mm lesion proximal to the PCI target vessel, which comprises only a small part of the entire coronary artery. It is possible that the artery studied did not reflect the extent of atherosclerosis present throughout the coronary arterial tree. Although we should assess NCLs from non-target vessels, it has been reported that MACE rates are similar in individual with and without CKD in their NCLs [24]. Third, IVUS examinations were performed only in patients with established angiographic CAD; therefore, the present analysis did not include patients with renal dysfunction who might not have coronary atheroma. Fourth, although lipid-rich plaques are considered vulnerable, plaque instability depends not only on an increased lipid volume, but also on the thinness of the cap, which can best be assessed using optical coherence tomography rather than IVUS. Therefore, a combination of several modalities, such as IVUS, optical coherence tomography and angioscopy should be tested for identifying vulnerable plaques in the future. Fifth, medications were not fixed during the study period. Changes in medications may have affected the results of this study. Sixth, we prescribed statin treatment to all patients, unless contraindicated. However, it is unclear whether intensive statin treatment was administered in this study, as medical therapy was left to the discretion of the treating physician [25]. We need a larger population study to verify the results of present study.

## Conclusion

Moderate to advanced CKD were associated with coronary plaque progression characterized by greater lipid and fibrotic plaque in NCL under statin treatment after culprit PCI.
